# How does mathematical modeling competency affect the creativity of middle school students? The roles of curiosity and guided inquiry teaching

**DOI:** 10.3389/fpsyg.2022.1044580

**Published:** 2023-01-13

**Authors:** Tian Wang, Libin Zhang, Zhiyong Xie, Jian Liu

**Affiliations:** ^1^Collaborative Innovation Center of Assessment Toward Basic Education Quality, Beijing Normal University, Beijing, China; ^2^China Education Innovation Institute, Beijing Normal University, Zhuhai, China; ^3^College of Teacher Education, South China Normal University, Guangzhou, China

**Keywords:** mathematical modeling competency, creativity, curiosity, guided inquiry teaching, middle school students

## Abstract

**Introduction:**

Mathematical modeling has become a crucial competence in mathematics education in many countries and regions due to the increasingly complex real-world problems that students face in the 21st century. Previous research has shown that mathematical modeling contributes to the development of students’ creativity, particularly with respect to stimulating and protecting the curiosity of children. However, previous studies have not explored or examined the relationships among middle school students’ mathematical modeling competency, curiosity, and creativity based on data drawn from large-scale assessments and have not investigated the influence of teachers’ teaching methods in this context.

**Methods:**

This study used convenience sampling to select 4,531 seventh-grade students from eastern and western, urban and rural areas in China. Online tests and questionnaires were used to measure their mathematical modeling competency, curiosity, creativity and guided inquiry teaching, and a moderated mediation model was used to analyze the effect of mathematical modeling competency on creativity.

**Results:**

The results showed the following. (1) There are statistically significant differences between boys and girls in terms of their mathematical modeling competency, curiosity, and creativity. Specifically, boys score significantly higher than girls on these variables. (2) Creativity exhibits a statistically significant positive correlation with mathematical modeling competency, curiosity, and guided inquiry teaching. (3) Curiosity mediates the relationship between mathematical modeling competency and creativity, and guided inquiry teaching moderates the influence of curiosity. In high-level guided inquiry teaching classes, curiosity has a stronger influence on creativity, and it mediates the relationship between mathematical modeling competency and creativity more strongly.

**Discussion:**

This study empirically verified the influence of mathematical modeling competency on creativity and provided a possible way to cultivate children’s creativity. Future research should use longitudinal analysis to verify the causal relationship between mathematical modeling competency and creativity and to systematically explore the possible path by which mathematical modeling competency affects creativity.

## 1. Introduction

Due to the continuous development of science and technology, mathematical modeling has come to play an increasingly important role in promoting social development and people’s ability to adapt to life, and mathematical modeling competency has become a key competency of future citizens (Kaiser, 2007). Since mathematics curriculum reform was initiated at the beginning of this century, many countries and regions have incorporated the cultivation of mathematical modeling competency or related modeling ideas and applications into mathematics curricula or teaching practices as an important goal of mathematics education (Blum et al., 2007; Cai, 2017) and have considered this skill to represent a necessary key competency for students in the 21st century (Cai and Xu, 2016). In the United States, the *Common Core State Standards for mathematics* list mathematical modeling as one of the eight Standards for Mathematical Practice so that students at all stages of learning can come to understand that mathematics can be used to solve problems in the real world (Common core state standards initiative, 2010). The description and requirements of mathematical modeling in China’s Mathematics Curriculum Standard undergo constant improvement (Huang et al., 2019). Mathematical modeling is a key component of literacy at the primary and middle school stages, and it guides students to learn from an early age that “mathematical models can be used to solve a class of problems and are the basic way to apply mathematics,” according to the *Mathematics Curriculum Standard for Compulsory Education* (The Ministry of Education of the People’s Republic of China, 2022).

Since mathematical modeling usually involves the task of solving unconventional and open problems in the real world, a task which requires creativity from the modeler to understand the real situation and propose new solutions (Niss and Blum, 2020), mathematical modeling is closely related to students’ creativity. In the face of the increasingly complex living and working environment of the 21st century, creativity has become an indispensable ability that allows people to cope with these new challenges and problems (OECD, 2010), and it has promoted the development of all aspects of society (Hennessey and Amabile, 2010). The China Education Innovation Institute of Beijing Normal University and the Twenty-First Century Learning (P21) of the United States incorporated “Creativity Competence” into *the 5Cs Framework for Twenty-first Century Key Competences* and claimed that new knowledge, new technology, new crafts and new values can be achieved *via* creativity (Gan et al., 2020). This concept can replace traditional resources, energy and capital as the driving force of sustainable economic development, and it emphasizes the fact that problem-solving based on real situations can enhance students’ creativity (Gan et al., 2020). In recent years, various international mathematical modeling activities have attached great importance to students’ creativity. The topics associated with modeling tasks cover cutting-edge fields such as “global warming,” “renewable energy” and “self-driving vehicles,” and mathematical modeling is used to stimulate and cultivate students’ creativity (Mei, 2018). Especially for primary and middle school students, the openness and uncertainty associated with mathematical modeling tasks can facilitate their development of creativity because they are full of curiosity and accustomed to creating (COMAP and SIAM, 2016).

Although correlations may exist between mathematical modeling and creativity, only a few studies have focused on this relationship (Wessels, 2014; Lu and Kaiser, 2022a), and there is a lack of large-scale evaluations based on empirical studies to verify these studies. This study aims to explore the path by which mathematical modeling competency influences the development of creativity by reference to large-scale evaluation data as well as to investigate the stimulation and cultivation of creativity in middle school students.

### 1.1. Mathematical modeling competency and creativity

Mathematical modeling is a cyclic process by which mathematics can be used to solve real problems, and it thus facilitates a two-way transformation between the mathematical world and the real world (Niss et al., 2007; Blum and Ferri, 2016). Mathematical modeling competency refers to the ability of a person to perform the required operations in a modeling environment to promote modeling (Niss et al., 2007) and is composed of the sub-competencies that are necessary to complete each step of the modeling cycle (Kaiser, 2007). A widely accepted model of the sub-competencies of mathematical modeling mainly includes five sub-competencies: simplifying, mathematising, working mathematically, interpreting and validating. Simplifying is the competency to understand real-world problems and develop real-world models; mathematising is the competency to establish mathematical models based on real-world models; working mathematically is the competency to solve mathematical problems in mathematical models; interpreting is the ability to interpret mathematical results in real-world models or situations; and validating is the competency to challenge the solution thus developed and to implement the modeling process again, if necessary (Maaß, 2006).

In a broad sense, creativity focuses mainly on everyday creativity, which refers the creative thinking in which everyone can engage in daily life and which can be improved *via* education and practice (OECD, 2019). Creativity denotes the ability of an individual to use relevant information and resources to produce novel and valuable ideas, programs, and products. It mainly includes three elements: creative personality, creative thinking and creative task engagement (Gan et al., 2020). Creative personality comprises the characteristics of curiosity, open mindedness, the courage to take on challenge and risks and independent self-confidence. Creative thinking comprises divergent thinking, convergent thinking and restructuring thinking, which are helpful when engaging in innovative activities. Creative task engagement involves participating and investing in practices that aim to produce novel and valuable results.

**Figure 1:**
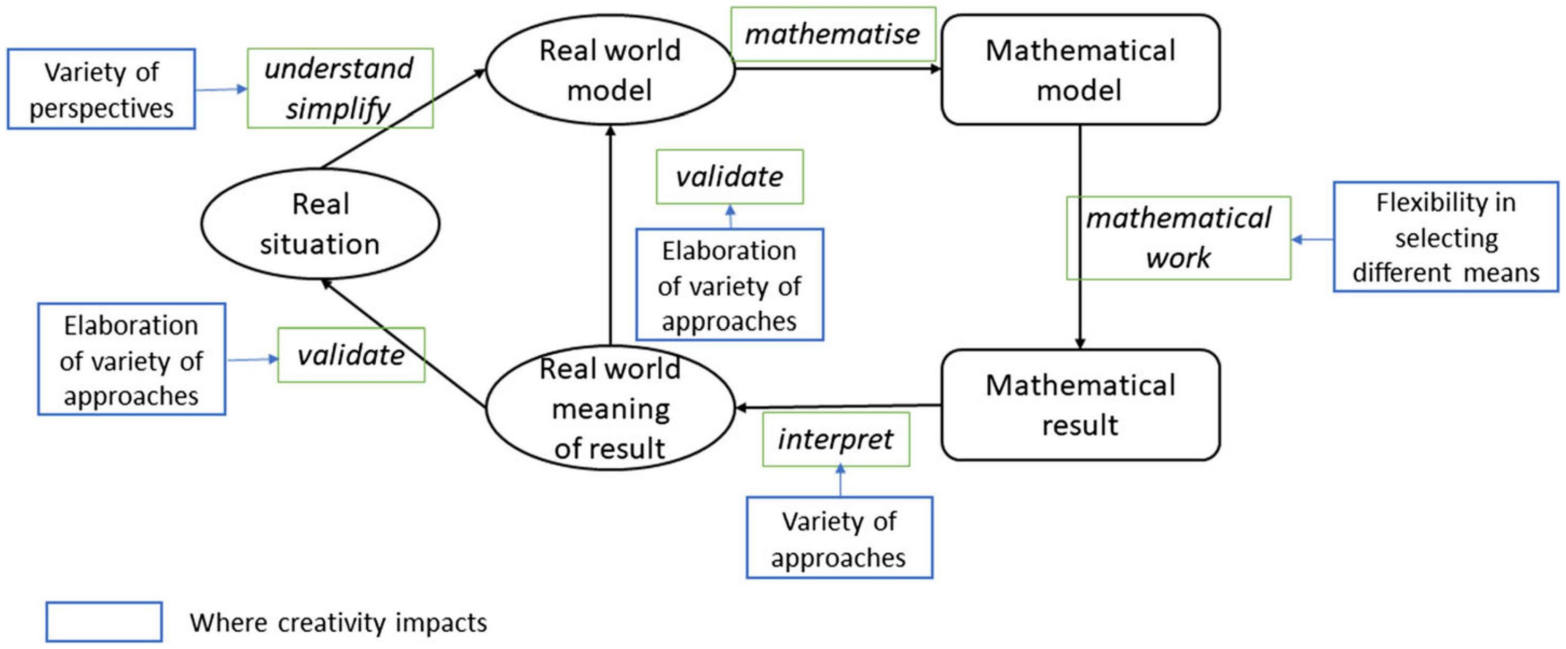
Modeling cycle enriched by aspects of creativity (Lu and Kaiser, 2022a) (CC-BY) (http://creativecommons.org/licenses/by/4.0/).

Mathematical modeling can effectively promote the development of students’ creativity and their mastery of mathematical knowledge and skills (Wessels, 2014). The development of creativity relies on mathematical tasks associated with higher levels of cognitive activity that are intended to stimulate students’ high-level cognitive processes (Leikin and Elgrably, 2020), and the characteristics of higher cognitive requirements for mathematical modeling tasks may help improve students’ creativity (Lu and Kaiser, 2022a). Lu and Kaiser (2022a) examined the process of mathematical modeling from the perspective of creativity and proposed a model of mathematical modeling cycle theory that includes creativity on the basis of the mathematical modeling process model (see Figure 1). According to this model, many processes involve creativity: in understanding and simplifying, the modeler analyzes a real situation from various perspectives, thereby generating various models of reality; in mathematical working, the modeler obtains the results of the mathematical model using various methods; in interpreting, the modeler interprets the mathematical results as results in the real world; and in validating, the modeler employs a variety of approaches to test the correctness of the results in a real-world situation. This model was applied to high school students, preservice mathematics teachers and in-service mathematics teachers. The results showed that mathematical modeling competency is significantly correlated with creativity (Wessels, 2014; Suh et al., 2017; Lu and Kaiser, 2022b). For high school students, the difficulty of modeling tasks may affect the relationship between mathematical modeling competency and creativity (Lu and Kaiser, 2022a). Wessels (2014) conducted a two-year longitudinal study to investigate preservice mathematics teachers. By analyzing all the materials (drafts, charts, formulas, etc.) involved in the process of solving mathematical modeling tasks, he found that the mathematical modeling process can effectively improve the individual’s level of creativity. Suh et al. (2017) selected two primary school teachers and their students in two classes as research objects. By reference to interviews with teachers, classroom observation and analysis of students’ works, these authors found that mathematical modeling can effectively improve students’ creativity, critical thinking, communication and collaboration.

Previous studies have provided evidence that creativity is involved in the mathematical modeling process (Lu and Kaiser, 2022a) and obtained certain empirical evidence in the context of teaching (Wessels, 2014; Suh et al., 2017). However, the potential relationship between mathematical modeling competency and creativity has rarely been explored by reference to large-scale assessments. It is necessary to verify the effectiveness of mathematical modeling for creativity cultivation based on empirical results. Simultaneously, the cultivation of children’s creativity has always received a great deal of attention in the field of educational psychology (Camp, 1994; Cheung et al., 2004; Hu et al., 2011). Researchers have found that the development of creativity in upper elementary school shows an upward trend; based on these findings, Smith and Carlsson proposed that the development of creativity may originate from high grades in primary school (Smith and Carlsson, 1990; Camp, 1994). Therefore, the impact of mathematical modeling on the creativity of primary and middle school students deserves further attention. In summary, **Research Hypothesis 1** of this study proposes that there is a significant positive correlation between the mathematical modeling competency of middle school students and their creativity.

### 1.2. Curiosity, mathematical modeling competency, and creativity

Curiosity is a ubiquitous psychological trait among humans. This term refers to one’s desire for learning even when the application of the knowledge is not readily apparent (Facione et al., 1994). Studies have shown that curiosity has a positive effect on individual creativity: the stronger an individual’s curiosity is, the greater that individual’s creativity (Celik et al., 2016; Hardy et al., 2017). The results of a meta-analysis of research regarding the relationship between curiosity and creativity indicated a moderate positive correlation between curiosity and creativity (Schutte and Malouff, 2020). According to the creative process model proposed by Mumford and McIntosh (2017), problem definition and information collection represent the initial steps of this process, and curiosity helps individuals collect information and define the problem that is to be solved (Schutte and Malouff, 2020). Hardy et al. (2017) analyzed the relationships among various types of curiosity, creative performance and creative problem-solving by reference to 122 college students and found that diversive curiosity (e.g., I find it interesting to learn new information) has a positive effect on creative performance *via* the complete mediation of the information gathering behavior associated with creative problem-solving. Simultaneously, as a positive emotion that provides motivation, curiosity may encourage new exploratory ideas, and behaviors (Fredrickson and Joiner, 2018), thereby enhancing individual creativity.

Mathematical modeling plays an important role in stimulating curiosity. From the perspective of cognitive neuroscience, curiosity is the result of situation-based prediction errors and information-based prediction errors (Gruber and Ranganath, 2019). On the one hand, when an individual is faced with a new or changing situation, a gap emerges between the prediction generated by his or her hippocampus and the actual situation at hand, which leads to exploratory behavior to address the associated uncertainty (O’Keefe and Nadel, 1979). On the other hand, when the knowledge that the individual wants to obtain is beyond that individual’s current level of knowledge or does not conform to the individual’s prior knowledge, an information gap is generated, thus triggering the individual’s curiosity (Litman, 2005; Gottlieb et al., 2013). By reference to classroom observations and interviews with teachers and students, Geiger et al. (2022) found that the openness and uncertainty exhibited by the real situation represent essential features of mathematical modeling tasks. Students may understand and analyze the situation they face in modeling tasks based on different sorts of previous experience and knowledge. The authenticity and diversity of the situations that are faced by students are quite different from those associated with ordinary mathematics problems, resulting in the occurrence of situational prediction errors that stimulate students’ curiosity. Simultaneously, mathematical modeling tasks are more challenging than traditional mathematics questions and thus require a higher level of cognition (English, 2021), so students are often unable to solve modeling tasks through simple memorization or the repetition of prior knowledge. Instead, it is necessary to integrate that prior knowledge with flexible applications based on *a priori* knowledge, thereby generating prediction errors regarding the information at hand and generating curiosity in students.

There is a strong positive correlation between individual curiosity and creativity (Celik et al., 2016; Hardy et al., 2017; Schutte and Malouff, 2020). The openness of mathematical modeling tasks and the high cognitive level required in this context may stimulate students’ curiosity (Litman, 2005; COMAP and SIAM, 2016; English, 2021), which may allow mathematical modeling to promote the development of creativity by enhancing individual curiosity. Further exploration of this path of influence may improve the theoretical model of creativity cultivation and promote the connection between mathematics learning and creativity cultivation. Since students’ curiosity may be stimulated during mathematical modeling processes and curiosity is an important predictor of creativity, curiosity may mediate the relationship between mathematical modeling competency and creativity. In summary, **Research Hypothesis 2** of this study proposes that curiosity mediates the relationship between middle school students’ mathematical modeling competency and their creativity.

### 1.3. Guided inquiry teaching, curiosity, and creativity

Guided inquiry teaching is a type of inquiry-based teaching. Inquiry-based teaching employs a student-centered approach in which teachers pose particular questions, such as open-ended or divergent questions, which allow students to respond in different ways (Oliveira, 2010). Inquiry-based teaching mainly includes the actions of making observations; asking questions; examining known information; planning surveys; reviewing known information based on experimental evidence; using tools to collect, analyze and interpret data; proposing answers; explaining and predicting; and exchanging results (National Research Council, 1996). In guided inquiry teaching, the source of the tasks or questions under consideration is the teacher, and the data collection methods and the interpretation of the results are designed and completed by the students; that is, the teacher provides the students with the questions that are to be investigated and any necessary information, while the students are required to design the inquiry program and develop a plan to solve and answer the problem by themselves (Blanchard et al., 2010; Chen et al., 2021). Studies have shown that inquiry-based teaching can effectively improve students’ academic performance (Schroeder et al., 2007; Minner et al., 2010) and has a positive impact on students’ learning attitudes and interests (Jiang and McComas, 2015).

Inquiry-based teaching may affect students’ curiosity and creativity as well as the relationship between those two factors. Erbas and Yenmez (2011) found that inquiry-based learning is positively correlated with curiosity, and Schijndel et al. (2018) found that students in an inquiry-based teaching experimental group exhibited greater curiosity than students in the control group. Rodríguez et al. (2019) found that inquiry-based learning can effectively promote the development of students’ creativity in controlled experiments. Guided inquiry teaching can stimulate students’ diversity of problem definition and information collection during the creative process, thus enabling students to generate new ideas. Studies have found that teaching methods or activities such as brainstorming and idea linking can affect the relationship between curiosity and creativity and that different teaching methods or activities may have different degrees of impact on the relationship between the two factors. Idea linking activities can promote students’ creativity more effectively than can brainstorming because they can establish connections with previous ideas (Hagtvedt et al., 2019). One characteristic of inquiry-based teaching is the generation of uncertainty (Schijndel et al., 2018), which is embodied in the possibility of collecting different information, using different methods, and obtaining different results. The uncertainty may affect the process by which the individual’s curiosity leads to the generation of new ideas, which may in turn affect the individual’s creativity. Loewenstein (1994), Choi et al. (2015), Yang et al. (2016), and Gruber and Ranganath (2019).

As a teaching method that stimulates curiosity and promotes creativity, guided inquiry teaching may regulate the relationship between students’ curiosity and creativity. Therefore, **Research Hypothesis 3** of this study proposes that guided inquiry teaching regulates the relationship between middle school students’ curiosity and their creativity.

### 1.4. The current study

**Figure 2:**
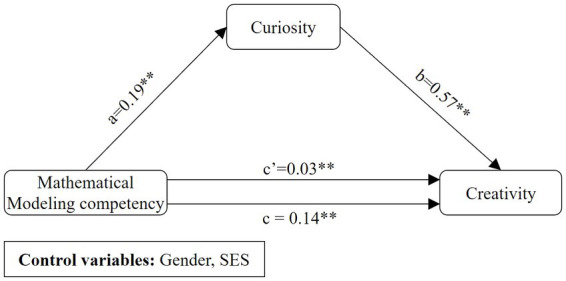
Hypothetical indirect pathways between mathematical modeling competency and creativity.

Based on previous studies, to explore the ways in which mathematical modeling competency influences creativity *via* curiosity as well as the influence of guided inquiry teaching on the relationship between curiosity and creativity, this study constructed a moderated mediation model of the influence of mathematical modeling competency on creativity (see Figure 2):

***Research Hypothesis 1***: The mathematical modeling competency of middle school students positively affects their creativity.

***Research Hypothesis 2***: Curiosity mediates the effect of mathematical modeling competency on the creativity of middle school students.

***Research Hypothesis 3***: Guided inquiry teaching regulates the relationship between middle school students’ curiosity and their creativity.

## 2. Materials and methods

### 2.1. Participants and data collection

This study used convenience sampling to select students from 83 middle schools, with 44 middle schools (53.0%) located in eastern China and 39 (47.0%) located in western China. The participants were 4,620 seventh-grade students who were enrolled in 2021; participants ranged from 11 to 13 years old at the time of the study. After removing incomplete data, we obtained 4,531 valid subjects, including 2,377 boys (52.5%) and 2,154 girls (47.5%).

In this study, data were collected using online tests and online questionnaires. All students who participated in the test entered the computer classroom and utilized the test platform under the guidance of the teacher. After starting the test, the students completed an online test of mathematical modeling competency (with a time limit of 40 min) and an online questionnaire (which had no time limit). Subsequently, the students answered an ungraded warm-up question to familiarize themselves with the various operations and answer specifications of the platform. The online test of mathematical modeling competency was to measure students’ mathematical modeling competency, and the online questionnaire was to measure students’ performance on curiosity, creativity and guided inquiry teaching.

### 2.2. Measures

#### 2.2.1. Online test of mathematical modeling competency

The online test of mathematical modeling competency included one warm-up question and two formal tasks, which were developed by the Beijing Normal University Regional Assessment of Education Quality (RAEQ). There were 15 items in the two formal tasks, which were choice items and open-ended items (see Appendix A). The assessment framework was based on the five sub-competencies model of mathematical modeling proposed by Kaiser (2007). The model was simplified and revised by using the thinking aloud and interviews of Chinese primary and middle school students to make it more suitable for their process of mathematical modeling. Finally, an assessment framework of mathematical modeling competency was developed, which includes four sub-competencies: understanding information, making models, working mathematically, and interpreting and validating. There were four items each for understanding information, making models and interpreting and validating, and three items for working mathematically. The RAEQ developed two modeling tasks for this online test based on the assessment framework, and the task situations were derived from *Mathematical Modelling: A Guidebook for Teachers and Teams* Galbraith and Holton (2018) and *Mathematical Modeling Handbook II: The Assessments* (Fletcher et al., 2013). After this adaptation had been completed, a 6-person thinking aloud session and external reviews by experts were used to examine the content validity of the test, and the pretest was used to examine its construct validity. Each open-ended item was coded by two or more raters and the interrater reliability scores were all more than 0.9. The test uses the IRT method to combine students’ scores on each item into an overall test score, which is standardized to produce a mathematical modeling competency score, in which context 500 is the average score and 100 is the standard deviation. The higher an individual’s score is, the higher that individual’s mathematical modeling competency. The confirmatory factor analysis results of the test were as follows: comparative fit index (CFI) = 0.931 > 0.90, Tucker-Lewis index (TLI) = 0.915 > 0.90, root mean square error of approximation (RMSEA) = 0.043 < 0.08, and standardized root mean square residual (SRMR) = 0.027 < 0.08 (Brown and Cudeck, 1993; Hox and Bechger, 1998; Kline, 2005). Cronbach’s alpha coefficient was 0.758, and the reliability and validity were acceptable.

#### 2.2.2. Creativity questionnaire

The creativity questionnaire was developed by the RAEQ and contains a total of 3 dimensions including 26 questions (see Appendix B). This questionnaire is based on the theoretical framework of creativity included in the 5Cs Framework for Twenty-First Century Key Competences and contains the three dimensions of creative personality, creative thinking, and creative task engagement (Gan et al., 2020). The questionnaire uses a five-point Likert scale to calculate the average score of each item as the creativity score (1 = Strongly disagree, 5 = Strongly agree). The higher this score is, the higher the individual’s level of creativity. This questionnaire was developed by reference to expert interviews, teacher interviews, and student pretests to ensure its validity. The Cronbach’s alpha coefficient of the creativity questionnaire was 0.937, which is acceptable. The results of the confirmatory factor analysis of the creativity questionnaire indicated that *x*^2^/*df* = 19.37, CFI = 0.922, TLI = 0.912, RMSEA = 0.064, and SRMR = 0.055.

#### 2.2.3. Curiosity questionnaire

The curiosity questionnaire is based on the adaptation of questions related to curiosity drawn from the California Critical Thinking Disposition Inventory (CCTDI) compiled by Facione et al. (1998). It contains a total of 5 items (see Appendix B). This questionnaire mainly examines respondents’ attitudes toward researching new things and their expectation of facing challenges. The questionnaire uses a five-point Likert scale to calculate the average of the scores of each item as the curiosity score (1 = Strongly disagree, 5 = Strongly agree). The higher this score is, the stronger the curiosity of the individual. This questionnaire underwent expert interviews, teacher interviews, and student pretests to ensure its validity. The Cronbach’s alpha coefficient of the curiosity questionnaire was 0.939, which is acceptable. The results of the confirmatory factor analysis of the curiosity questionnaire indicated that *x*^2^/*df* = 10.03, CFI = 0.998, TLI = 0.995, RMSEA = 0.045, and SRMR = 0.006.

#### 2.2.4. Guided inquiry teaching questionnaire

The guided inquiry teaching questionnaire was developed by the RAEQ and contains a total of 5 items (see Appendix B). The project team developed a questionnaire based on the characteristics of the guided inquiry teaching process proposed by Blanchard et al. (2010). The questionnaire used a five-point Likert scale to calculate the average of the scores of each item as the guided inquiry teaching score (1 = Strongly disagree, 5 = Strongly agree). The higher this score is, the more strongly the individual feels that the teacher used guided inquiry teaching. This questionnaire underwent expert interviews, teacher interviews, and student pretests to ensure its validity. The Cronbach’s alpha coefficient of the curiosity questionnaire was 0.946, which is acceptable. The results of the confirmatory factor analysis of the guided inquiry teaching questionnaire indicated that *x*^2^/*df* = 7.37, CFI = 0.999, TLI = 0.997, RMSEA = 0.037, and SRMR = 0.004.

#### 2.2.5. Demographic variables

This study mainly investigated demographic variables such as the gender and family socioeconomic status (SES) of students. Among these variables, SES was assessed using the relevant part of the PISA 2012 technical report of OECD (2014), which is mainly divided into three parts: parents’ level of education, highest occupational status, and family possessions. The level of education primarily refers to the highest level of education attained by the parents of the surveyed student, and the highest level of education attained by the parents is regarded as the “parents’ level of education” of the student. The highest occupational status of the parents refers to the main job of the parents of the surveyed student, and the highest occupational status of the parents is regarded as the “parental highest occupational status” of the student. Family possessions are measured mainly in terms of four aspects: family wealth, cultural possessions, family education resources, and family books. Finally, the standardized scores of the three components of SES are used as composite scores of the students’ SES.

## 3. Results

### 3.1. Common method biases analysis

The Harman single-factor test was used (Malhotra et al., 2006) to evaluate common method bias (Podsakoff et al., 2003). We conducted exploratory factor analysis to investigate all 36 items related to creativity, curiosity and guided inquiry teaching using SPSS 28 software (SPSS Inc., Chicago, Illinois, United States). The results showed that Bartlett = 113974.69, *df* = 630, *p* < 0.01, KMO = 0.963, communalities variance = 69.56%, the total variance explained by the first common factor was 19.16%, i.e., less than the critical value of 40% (Tang and Wen, 2020). Therefore, no common method bias affected the results of the current study.

### 3.2. Descriptive statistics and correlation analysis

Prior to the formal analysis, all main variables were tested for normality, and the results showed that all variables had kurtosis values between −0.339 and 0.618 and skewness values between −0.957 and 0.220, indicating that all variables followed a normal distribution. Table 1 shows the means (M), standard deviations (SDs), gender-based differences, and intercorrelations among the key variables. Children’s mathematical modeling competency, curiosity, and guided inquiry teaching were significantly and positively related to their creativity (*r* = 0.149 ~ 0.586, *p*_s_ < 0.01). Both children’s mathematical modeling competency and their curiosity were positively correlated with guided inquiry teaching (*r* = 0.167, 0.507, *p*_s_ < 0.01). The correlation between children’s mathematical modeling competency and curiosity was also significant (*r* = 0.192, *p*_s_ < 0.01). In addition, independent-sample t tests indicated significant gender differences in mathematical modeling competency, creativity, and curiosity; specifically, boys scored significantly higher than girls on those variables.

**Table 1:** Means, SDs, gender difference, and intercorrelations among key variables.

	1	2	3	4	5	6
1. Gender	1					
2. SES	0.023	1				
3. Mathematical modeling competency	0.035*	0.040**	1			
4. Creativity	0.104**	0.084**	0.149**	1		
5. Curiosity	0.071**	0.048**	0.192**	0.586**	1	
6. Guided inquiry teaching	0.009	0.031*	0.167**	0.440**	0.507**	1
M ± SD (the whole sample)	–	–	483.57 ± 99.61	3.72 ± 0.71	3.91 ± 0.94	4.12 ± 0.89
M ± SD (Boys)	–	–	486.9 ± 103.1	3.79 ± 0.74	3.97 ± 0.97	4.12 ± 0.92
M ± SD (Girls)	–	–	479.9 ± 95.53	3.64 ± 0.66	3.83 ± 0.90	4.11 ± 0.87
t			2.39	7.06	4.85	0.59
*p*			0.017	<0.001	<0.001	0.554

*p < 0.05; **p < 0.01.

### 3.3. The mediating effect of curiosity

**Figure 3:**
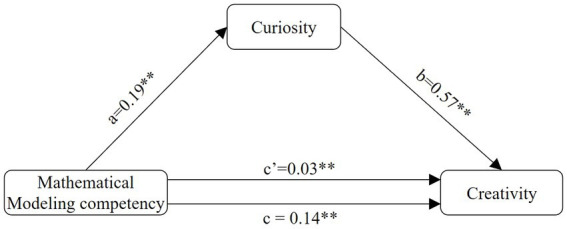
The mediating effect of curiosity on mathematical modeling competency and creativity.

To examine the mediating effect of mathematical modeling competency on creativity *via* curiosity, we computed 95% confidence intervals (95% CIs) using the bootstrap method with 5,000 replications with the help of the PROCESS 4.0 plug-in (Model 4). After controlling for gender and SES, mathematical modeling competency significantly positively predicted curiosity (*β* = 0.19, *p* < 0.001). As shown in Figure 3, curiosity significantly positively predicted creativity (*β* = 0.57, *p* < 0.001), and mathematical modeling competency had a significant direct effect on creativity (*β* = 0.03, *p* = 0.004). The 95% confidence interval of the bootstrap mediation effect did not include zero (effect size = 0.108, 95% CI [0.092, 0.126], accounting for 78.57% of the total effect), thus indicating that mathematical modeling competency has a significant mediating effect on creativity.

### 3.4. The moderating effect of guided inquiry teaching

To test Hypothesis 3, which pertained to the moderating effect of guided inquiry teaching on the influence of curiosity on creativity, we used Model 14 in PROCESS 4.0 to conduct the relevant analysis. As shown in Table 2, after controlling for gender and SES, curiosity and guided inquiry teaching significantly positively predicted creativity (*β* = 0.49, 0.20, *p* < 0.001), and the interaction between guided inquiry teaching and curiosity was significantly related to creativity (*β* = 0.03, *p* = 0.004). Thus, the results confirm our hypothesis that guided inquiry teaching moderates the relationship between curiosity and creativity.

**Table 2:** The moderating effect of guided inquiry teaching.

	Creativity		
	*β*	SE	*t*
Gender	0.121	0.024	5.139**
SES	0.002	0.000	4.570**
Mathematical modeling competency	0.022	0.012	1.869
Curiosity	0.478	0.014	34.785**
Guided inquiry teaching	0.203	0.014	14.455**
Guided inquiry teaching * curiosity	0.031	0.010	3.074**
*R* ^2^	0.380		
*F*	461.796**		

**p < 0.01.

**Figure 4:**
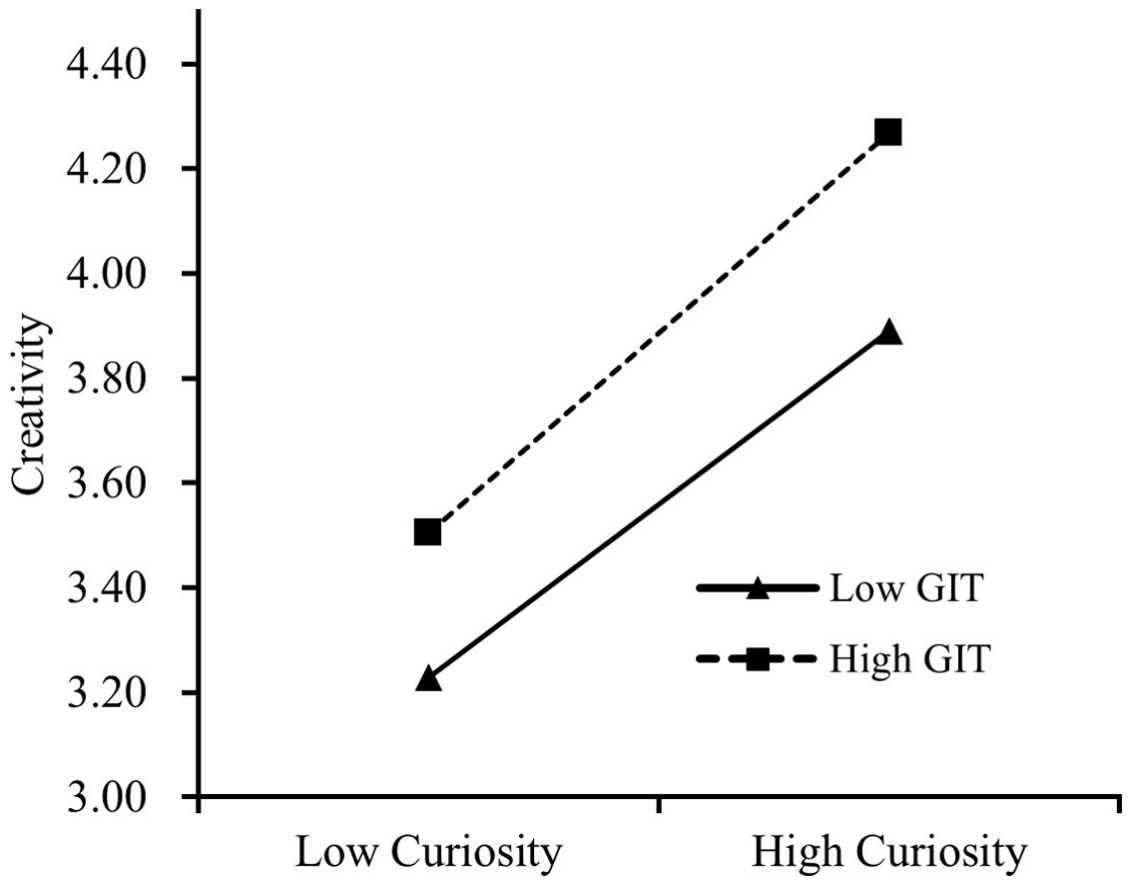
Simple slope test.

In addition, to improve our understanding of this interaction, we plotted the simple slope (Figure 4), and the results of the simple slope test show that in the case of high levels of guided inquiry teaching (M + 1SD), the slope is *B* = 0.51, *p* < 0.001, while in the case of low levels of guided inquiry teaching (M–1SD), the slope is *B* = 0.44, *p* < 0.001, thus indicating that curiosity has a stronger positive impact on creativity in the high guided inquiry teaching condition. Mathematical modeling competency also had a stronger mediating effect on creativity *via* curiosity in this condition (effect size = 0.10, 95% CI [0.08, 0.11]).

## 4. Discussion

### 4.1. Mathematical modeling positively affects the creativity of middle school students

This study found that the mathematical modeling of middle school students plays a positive role in their development of creativity; accordingly, Research Hypothesis 1 was verified. The stronger a student’s mathematical modeling competency is, the higher that student’s level of creativity. To a certain extent, these research results support the view that mathematical modeling helps improve the development of students’ creativity (Wessels, 2014; Suh et al., 2017; Lu and Kaiser, 2022a). The modeling cycle, which is enriched by aspects of creativity proposed by Lu and Kaiser (2022a) (Figure 1), explains that students may generate a variety of new ideas during each step of mathematical modeling and may take different approaches to the task of solving problems, thus providing support for the development of the divergent thinking of students. Simultaneously, children exhibit a high level of natural enthusiasm for mathematics and are skilled at creativity, and so the open nature of mathematical modeling tasks can stimulate their creativity and choice (COMAP and SIAM, 2016). Therefore, teachers can use the uncertainty and openness of the mathematical modeling task in mathematics classrooms to guide and cultivate middle school students’ divergent thinking, not merely by focusing on the knowledge and skills they need to solve practical problems but also by emphasizing and developing their creativity, which may be reflected in the modeling process.

### 4.2. Curiosity mediates the relationship between mathematical modeling competency and creativity

This study found that curiosity partially mediates the effect of the mathematical modeling competency of middle school students on creativity; accordingly, Research Hypothesis 2 was verified. Mathematical modeling plays a positive role in the development of individual curiosity. The stronger students’ mathematical modeling competency is, the greater their curiosity is, which to some extent verifies the theory that curiosity originates from situation-based prediction errors and information-based prediction errors (Gruber and Ranganath, 2019). Based on Lu and Kaiser’s (2022a) theoretical framework, the different perspectives and approaches used by students in solving mathematical modeling tasks may be the source of their curiosity. The mathematical modeling tasks used in this study are mainly based on personal and scientific situations, which are more realistic and feature more uncertainty than situations in which students solve problems in ordinary mathematics education. Errors trigger students’ desire to explore different content in the situation at hand, thereby inspiring students’ curiosity. Simultaneously, solving modeling tasks requires students to understand and collect relevant information, construct appropriate mathematical models independently, solve models, and explain and verify the solutions to realistic problems, a process which differs from the “standard process” used by students to solve conventional mathematical problems. Mathematical modeling tasks stimulate prior knowledge and information prediction errors between the process and reality, thereby enhancing students’ creativity. For example, students collected different useful information to make different mathematical models in the example item (see Appendix A). Some of them chose “Use 1 liter of water every time” so that they make a more complete mathematical models and the others make the different mathematical models. The uncertainty of the information collection could promote the motivation of students to further explore different approaches to solve the problem.

On the other hand, curiosity has a positive impact on individual creativity. Middle school students with higher levels of curiosity tend to exhibit higher levels of creativity, a finding which supports the conclusions of previous studies (Celik et al., 2016; Hardy et al., 2017; Schutte and Malouff, 2020). According to Mumford and McIntosh (2017) creative thinking process model, problem definition and information collection are the initial steps in this process. On this basis, students are given the opportunity to understand the task situation and collect relevant data and information independently, which helps them participate in the process of creative thinking and enhance their creativity.

Based on the mediation path of “mathematical modeling competency-curiosity-creativity,” teachers can focus on the following two strategies in their daily teaching using mathematical modeling. First, teachers should focus on guiding students to understand and explore realistic problems and situations and helping students consider the uncertainty involved in the task at hand. When middle school students face realistic problems, due to their limited knowledge of the real world, teachers must guide students to consider ways of understanding the problem situation and the possibilities that exist in this context. Teachers should help students understand the uncertainty involved in real situations as well as ways of using mathematics to solve real problems with the aim of effectively stimulating and cultivating students’ curiosity and creativity. Second, when selecting and designing modeling tasks, teachers should control the difficulty and complexity of such tasks. The cognitive development of middle school students is not yet fully mature, and their mathematical knowledge and skills remain limited. Therefore, the realistic problems selected by teachers should enable students to understand the situation at hand and allow them to try to develop solutions; accordingly, these problems should not be too simple and routine to stimulate students’ high-level cognitive processes, nor should they be too difficult and complex, thus causing students to lose their motivation and interest in inquiry.

### 4.3. Guided inquiry teaching moderates the relationship between curiosity and creativity

This study found that the influence of middle school students’ curiosity on creativity is moderated by guided inquiry teaching; accordingly, Research Hypothesis 3 was validated. The impact of curiosity on creativity is higher in individuals who perceive a higher level of guided inquiry teaching than in individuals who perceive a lower level of guided inquiry teaching. In other words, guided inquiry-based teaching can promote the positive impact of middle school students’ curiosity on their creativity. One possible explanation for this influence is related to the free exploration space provided by guided inquiry teaching; one characteristic of guided inquiry teaching is that after teachers provide students with tasks and the necessary explanations, students must independently design their own process of inquiry and approach to problem-solving (Blanchard et al., 2010; Chen et al., 2021), thus offering students the freedom to play and operate in this context. In a classroom in which teachers use more guided inquiry teaching, students may analyze and solve problems from additional perspectives, and they may have more opportunities to try multiple strategies and solutions; accordingly, their curiosity is more likely to promote the development of creativity (Zhao, 2018).

Based on the positive effect of guided inquiry teaching on the relationship between curiosity and creativity, teachers should consider the positive impact of teaching methods and classroom climate (Lucas and Spencer, 2017). In China, demonstration or lecturing are mostly applied by primary and middle school teachers in class, leading to students having few opportunities to think independently (Crehan, 2016). The results provide a suggestion that Chinese teachers should play guiding roles in students’ learning processes and give students more freedom in inquiry. Teachers should avoid excessive participation that might reduce the effectiveness of students’ creativity, thereby effectively promoting students’ curiosity and creativity.

## 5. Limitations

This study faced certain limitations that should be addressed in future studies. First, this study adopted a cross-sectional research design, and in the future, it is necessary to investigate the causal relationship between mathematical modeling and creativity in further detail using experimental studies or longitudinal tracking studies. Second, mathematical modeling competency includes not only the sub-competencies of the modeling process but also metacognitive modeling competencies and other elements. In the future, researchers can use log data to measure metacognitive competency and incorporate it into mathematical modeling competency. Third, the influence of mathematical modeling competency on creativity *via* curiosity is only one of the possible paths associated with this relationship, and there may be other factors that mediate this relationship. In the future, additional empirical studies are necessary to verify the influence paths highlighted by this study, and more variables should be used to explore the possible factors mediating this relationship to provide more theoretical support for the cultivation of creativity. Moreover, students’ self-reports may exhibit certain biases, and other, more objective methods should be used to measure relevant variables in the future.

## 6. Conclusion

In conclusion, this study expands our understanding of the relationship between mathematical modeling competency and creativity and explores the role of curiosity as a mediator and that of guided inquiry teaching as a moderator in this relationship. This model enriches the existing theories on the relationship between mathematical modeling competency and creativity and improves the theoretical basis for teachers to use mathematical modeling tasks and guided inquiry teaching to cultivate students’ creativity. The results of this study were as follows. (1) Creativity can be influenced by middle school students’ mathematical modeling competency. (2) Mathematical modeling can promote the creativity of middle school students by stimulating their curiosity. (3) Guided inquiry teaching can improve the impact of middle school students’ curiosity on their creativity. Compared with low levels of guided inquiry teaching, high levels of guided inquiry teaching can improve the positive effect of curiosity on creativity.

## Data availability statement

The original contributions presented in the study are included in the article/supplementary material, further inquiries can be directed to the corresponding author.

## Ethics statement

The studies involving human participants were reviewed and approved by the Institutional Review Board (IRB) at the Collaborative Innovation Center of Assessment toward Basic Education Quality at Beijing Normal University. Written informed consent to participate in this study was provided by the participants’ legal guardian/next of kin.

## Author contributions

TW: writing—original draft and review, writing—editing, and data—analysis. LZ: writing—original draft and review and data—analysis. ZX: writing—review and writing—editing. JL: writing—editing, supervision, and project administration. All authors contributed to the article and approved the submitted version.

## Funding

This work was supported by the Collaborative Innovation Center of Assessment Toward Basic Education Quality, Beijing Normal University [Grant number BJZK-2021A1-20016].

## Conflict of interest

The authors declare that this research was conducted in the absence of any commercial or financial relationships that could be construed as potential conflicts of interest.

## Publisher’s note

All claims expressed in this article are solely those of the authors and do not necessarily represent those of their affiliated organizations, or those of the publisher, the editors and the reviewers. Any product that may be evaluated in this article, or claim that may be made by its manufacturer, is not guaranteed or endorsed by the publisher.
