# Supplementary material for: How does mathematical modeling competency affect the creativity of middle school students? The roles of curiosity and guided inquiry teaching
Source: Front Psychol. 2023 Jan 13;13:1044580. doi: 10.3389/fpsyg.2022.1044580 (PMC9880854; doi:10.3389/fpsyg.2022.1044580)
Supplement: Supplementary file 1 [file Presentation_1.pdf]

Appendix A: The example item of the brushing tasks

The description of the task: Recently, a report as shown in the picture appeared on the Internet. Do you think the report is true? Please check it out!

A family of four wastes around 450 liter of water a week if the faucet is left running while brushing their teeth.

Please collect the information that is useful for solving the problem.

A. Everyone should brush their teeth twice a day.

B. The mouthwash cup has a capacity of 500 ml.

C. Brush teeth for an average of three minutes.

D. Use 1 cm of toothpaste every time.

E. The length of the toothbrush is 18 centimeters.

F. 3 liters of water come out of the faucet every minute.

G. The water temperature is about 25 ℃.

H. Use 1 liter of water every time.

Appendix B: The example items of online questionnaire

Creativity questionnaire

(1) When solving daily problems, I always find new uses for the objects around me.

A. Strongly disagree B. Disagree C. Don't know D. Agree E. Strongly agree

(2) I often publicly express opinions that differ from the majority.

A. Strongly disagree B. Disagree C. Don't know D. Agree E. Strongly agree

(3) Faced with challenges, I always ask myself to use innovative ways or methods to solve problems.

A. Strongly disagree B. Disagree C. Don't know D. Agree E. Strongly agree

Curiosity questionnaire

(1) Studying new things can enrich my life.

A. Strongly disagree B. Disagree C. Don't know D. Agree E. Strongly agree

(2) I am looking forward to facing challenging things.

A. Strongly disagree B. Disagree C. Don't know D. Agree E. Strongly agree

(3) Solving difficult problems is fun.

A. Strongly disagree B. Disagree C. Don't know D. Agree E. Strongly agree

Guided inquiry teaching questionnaire

(1) The teacher led us into a discussion on a problem.

A. Strongly disagree B. Disagree C. Don't know D. Agree E. Strongly agree

(2) The teacher encouraged us to make guesses and test them or come to conclusions through various methods.

A. Strongly disagree B. Disagree C. Don't know D. Agree E. Strongly agree

(3) The teacher encouraged us to think differently about solving problems.

A. Strongly disagree B. Disagree C. Don't know D. Agree E. Strongly agree
